# Integrins regulation of wound healing processes: insights for chronic skin wound therapeutics

**DOI:** 10.3389/fcimb.2024.1324441

**Published:** 2024-03-05

**Authors:** Dong Yu, Zhaoyu Lu, Fengsong Nie, Yang Chong

**Affiliations:** ^1^ Department of Traditional Chinese Medicine, The Affiliated Hospital of Yangzhou University, Yangzhou University, Yangzhou, Jiangsu, China; ^2^ Department of General Surgery, The Affiliated Hospital of Yangzhou University, Yangzhou University, Yangzhou, Jiangsu, China

**Keywords:** integrin, wound healing, wound chronicity, bacterial infection, targeted therapy

## Abstract

Integrins are heterodimers composed of non-covalently associated alpha and beta subunits that mediate the dynamic linkage between extracellular adhesion molecules and the intracellular actin cytoskeleton. Integrins are present in various tissues and organs and are involved in different physiological and pathological molecular responses *in vivo*. Wound healing is an important process in the recovery from traumatic diseases and consists of three overlapping phases: inflammation, proliferation, and remodeling. Integrin regulation acts throughout the wound healing process to promote wound healing. Prolonged inflammation may lead to failure of wound healing, such as wound chronicity. One of the main causes of chronic wound formation is bacterial colonization of the wound. In this review, we review the role of integrins in the regulation of wound healing processes such as angiogenesis and re-epithelialization, as well as the role of integrins in mediating bacterial infections during wound chronicity, and the challenges and prospects of integrins as therapeutic targets for infected wound healing.

## 1 Introduction

**Figure 1:**
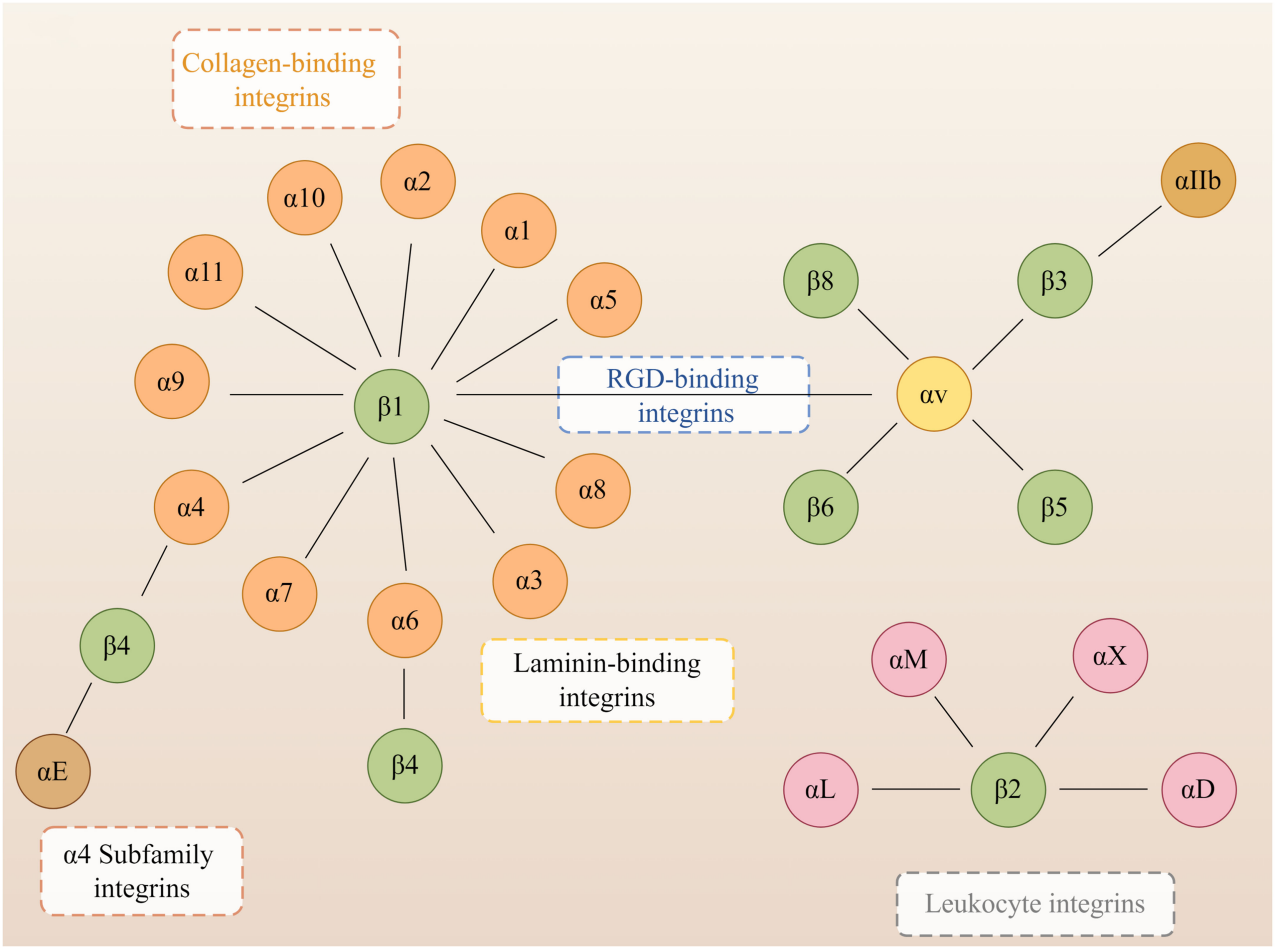
Members of the human integrin superfamily. At least 18 α subunits and eight β subunits have been identified in humans, and they can produce 24 different integrins. The integrin subunits that bind to each other to form heterodimers are connected by solid lines. By Figdraw.

Integrins are heterodimers composed of non-covalently associated α and β subunits that link the extracellular matrix (ECM) to the cytoskeleton and mediate dynamic connections between extracellular adhesion molecules and the intracellular actin cytoskeleton as well as intermediate filaments (Hynes, 2004). Intracellular proteins that bind to the cytoplasmic tail of integrins regulate the binding of integrins to extracellular ligands and integrin localization and transport. Cytoplasmic integrin-binding proteins also function downstream of integrins, mediating connections to the cytoskeleton and signaling cascades that affect cell motility, growth, and survival (Morse et al., 2014). In mammals, integrins are composed of 18 α and eight β subunits, classified into laminin-binding integrins (**Figure 1**): α1β1, α2β1, α3β1, α6β1, α7β1, and α6β4, collagen-binding integrins: α1β1, α2β1, α3β1, α10β1, and α11β1, leukocyte integrins: αLβ2, αMβ2, αXβ2, and αDβ2 and RGD-recognizing integrins: α5β1, αVβ1, αVβ3, αVβ5, αVβ6, αVβ8, and αIIbβ3, and with different binding properties and different tissue distribution (Takada et al., 2007). Integrins are involved in various bodily processes, including trauma, immunity, infection, cell proliferation, inflammation, angiogenesis, and tumors (Hostetter, 1996; LaFlamme and Auer, 1996; Desgrosellier and Cheresh, 2010; Mezu-Ndubuisi and Maheshwari, 2021).

**Figure 2:**
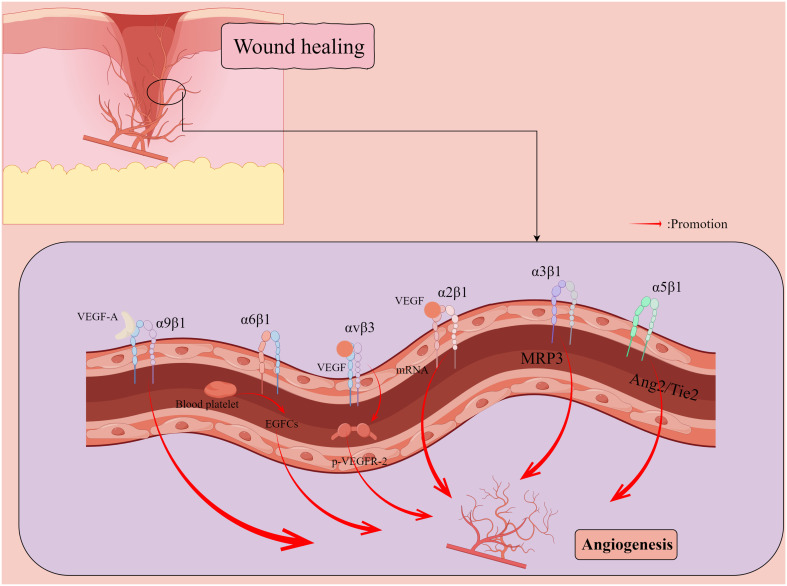
Promotion of new capillary formation by integrins during wound healing. Vascular endothelial growth factor (VEGF) induces a 5- to 7-fold increase in the protein expression of two collagen receptors, α1β1 and α2β1 integrins, on the surface of dermal microvascular endothelial cells (ECs) through the induction of mRNAs encoding α1 and α2 integrins subunits. α5 integrin localizes to cell junctions and participates in the angiopoietin (Ang)/Tie2 signaling pathway to maintain vascular homeostasis. αvβ3 integrin synergizes with VEGF to activate angiogenesis in ECs through VEGFR-2 phosphorylation. α6β1 integrin appears to promote platelet pro-mediated angiogenesis associated with endothelial colony forming cells (ECFCs). VEGF-A can induce endothelial and cancer cell migration by directly binding α9β1 integrin. By Figdraw.

**Figure 3:**
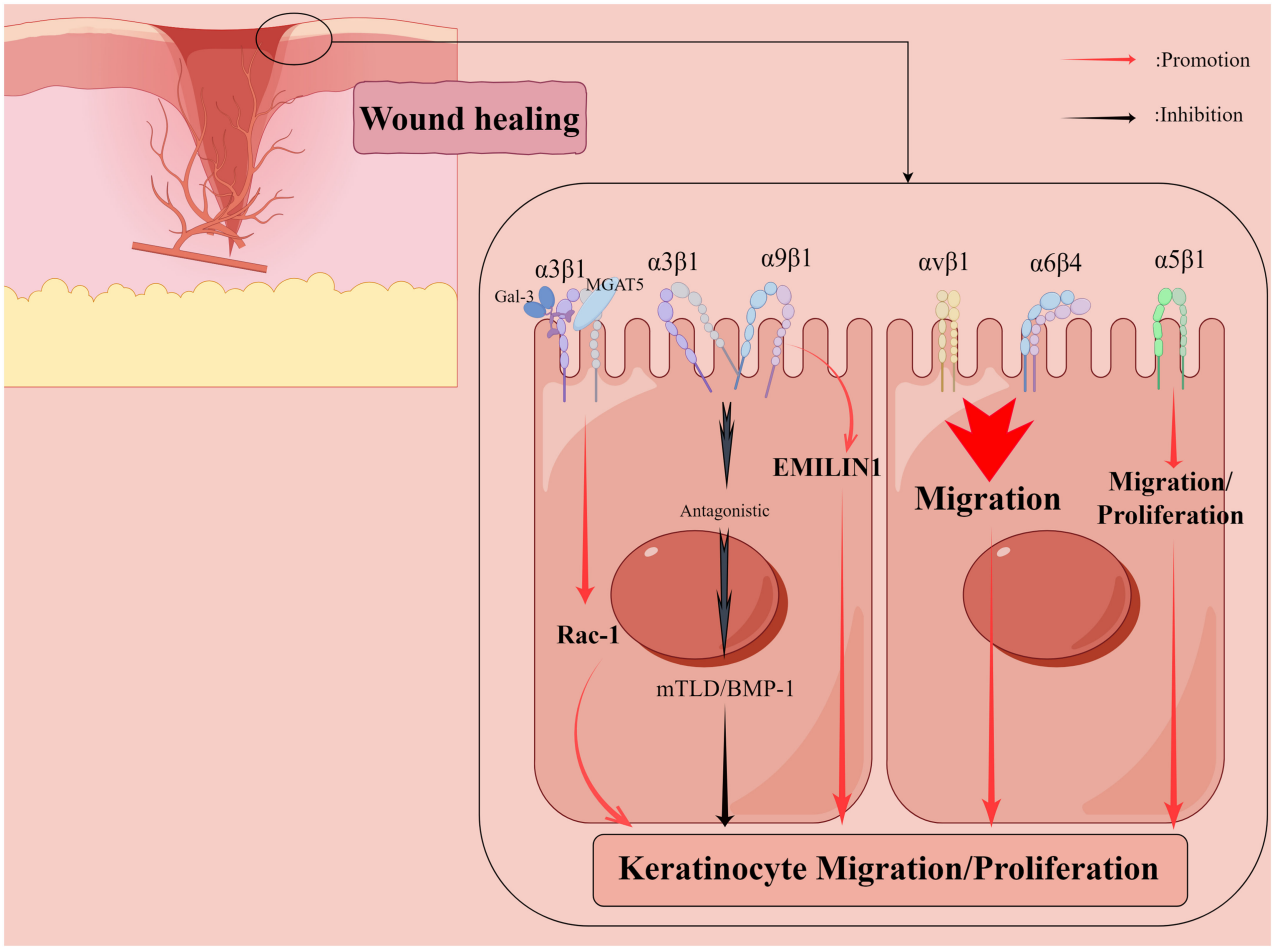
Shows that integrins regulate the re-epithelialization phase of the wound healing process. Galectin-3 promotes epithelial cell migration by cross-linking Mannoside Acetylglucosaminyltransferase 5 (MGAT5)-modified complex N-glycans on α3β1 integrins and subsequently activating α3β1-integrin-Rac1 signaling to promote lamellar pseudopod formation. The interaction of α5β1 integrins with fibronectin may contribute to keratinocyte proliferation in addition to promoting keratinocyte adhesion and motility on this matrix. α9β1 integrin interacts with another ECM component, elastic microfibril interface localization protein 1 (EMILIN1), to regulate keratinocyte proliferation, but α9β1 integrin antagonizes α3β1-dependent mTLD/BMP-1 expression and skin basement membrane reorganization and maturation. αvβ1 and α6β4 integrins also regulate keratinocyte migration. By Figdraw.

Skin wounds, in the context of successful healing, include dynamic processes in three overlapping phases: inflammation, proliferation, and tissue remodeling (Martin, 1997). Wound repair is tightly regulated by many factors, including cell-ECM interactions (Martin, 1997), growth factors, and matrix metalloproteinases (MMP) (Gál et al., 2017). The integrin family regulates all processes of wound healing (**Table 1**), such as hemostasis, inflammation, angiogenesis (**Figure 2**), re-epithelialization (**Figure 3**), and fibrosis. Disruption of these regulatory mechanisms at any stage can lead to chronic or non-healing wounds where factors such as persistent inflammation and impaired barrier (Brem and Tomic-Canic, 2007; Harding et al., 2002), oxygenation response (Bishop, 2008), bacterial infection (Edwards and Harding, 2004), age (Swift et al., 2001), and disease state (Brem and Tomic-Canic, 2007) can impede the skin’s ability to repair wounds effectively. In reality, chronic wounds are often accompanied by bacterial infections, and some bacteria, such as *Staphylococcus aureus (S. aureus)* and *Pseudomonas aeruginosa (P. aeruginosa)*, can mediate the integrin family to promote the formation of chronic wounds and thus cause them to persist (Edwards and Harding, 2004; Canchy et al., 2023). Abnormal wound healing is a major challenge in the treatment of skin wounds, and chronic wounds pose a serious emotional and financial burden to patients (Olsson et al., 2019). In this review, we review the role of integrins as bridges in bacterial-cell interactions in the context of wound healing and assess the role of integrins as nodes to inhibit bacteria in wound chronicity, as well as the challenges and perspectives of integrins as targets for therapeutic wound healing.

**Table 1:** Main secretory sites and functional roles of different types of integrins.

Type	Ligands	Secretion sites	Functional roles
**α_1_β_1_**	Laminin, collagen	EC, FBL, monocytes, macrophages, and myofibroblasts	Mediating VEGF-driven angiogenesis, negative feedback regulation of collagen synthesis in FBL (Senger and Davis, 2011; Gardner et al., 1999; Senger et al., 1997)
**α_2_β_1_**	Laminin, collagen	Platelets, KC, EC and FBL	Mediates KC migration and VEGF-driven angiogenesis (Senger et al., 1997; Grenache et al., 2007)
**α_3_β_1_**	Laminin, platelet-reactive protein	KC、EC and FBL	Regulation of KC migration during re-epithelialization (Margadant et al., 2009), control of angiogenesis and TGF-β1-mediated responses (da Silva et al., 2010)
**α_4_β_1_**	Thrombospondin, fibronectin, bone bridge protein, ADAM, EDA, VCAM, etc (Huhtala et al., 1995; Shinde et al., 2015; Abonia et al., 2006)	Leukocytes, FBL, and EC	Regulation of FBL proliferation and TGF-β1 processing (Shinde et al., 2015)
**α_5_β_1_**	Fibronectin, bone bridging protein, pro-fibronectin, ADAM, CCN, etc (Huhtala et al., 1995; Lau, 2016)	Platelets, KCs, ECs, FBLs	Promote KC migration (Di Russo et al., 2021), etc.
**α_6_β_1_**	Laminin, coagulation-reactive protein, Cyr61, CCN, etc (Lau, 2016)	Platelets, EC, leukocytes, and FBL	may be involved in platelet-vessel wall interactions and angiogenesis (Huang et al., 2016); interaction with CCN1/Cyr61 promotes myofibroblast senescence and controls fibrogenesis (Jun and Lau, 2010)
**α_7_β_1_**	Laminin	Expressed by muscle cells, vascular smooth muscle cells, etc (Riederer et al., 2015; Burkin and Kaufman, 1999)	
**α_8_β_1_**	FN, TGF-β1, etc.	Myofibroblasts	Lead to fibrotic reaction (Bouzeghrane et al., 2004)
**α_9_β_1_**	EDA-FN, VEGF, etc (Shinde et al., 2015; Eto et al., 2002; Vlahakis et al., 2005)	KCs, FBLs, neutrophils, and ECs	Regulation of KC and FBL growth, neutrophil chemotaxis, and EC migration and angiogenesis (Nakayama et al., 2010; Oommen et al., 2011; Høye et al., 2012)
**α_10_β_1_**	Collagen	FBL	May mediate the adhesion of FBL to collagen and dynamic connective tissue remodeling events (Zeltz and Gullberg, 2016)
**α_11_β_1_**	Collagen	FBL	Controls myofibroblast differentiation and may mediate adhesion of FBL to collagen and contribute to collagen reorganization (Zeltz and Gullberg, 2016)
**α_v_β_1_**	FN, TGF-β1, etc.	KC、EC	Mediating KC adhesion during re-epithelialization (Jakhu et al., 2018)
**α_v_β_3_**	Fibronectin(pro), FGF-2, TGF-β1, CCN1/Cyr6, CCN2/CTGF and CCN3/NOV, etc (Lau, 2016; Rusnati et al., 1997; Lin et al., 2005)	EC, platelets, FBL, and macrophages	Required for neoangiogenesis; regulates fibronectin network structure and stability; mediates EC adhesion to CCN1/Cyr6 and CCN2/CTGF; EC survival; pericyte retention in the vasculature; and FBL proliferation (Mitchell et al., 2009)
**α_v_β_5_**	TGF-β1, VEGF, CCN1/Cyr6, CCN3/NOV, etc (Lau, 2016; Lin et al., 2005)	EC, FBL, and Skin KC	may be involved in the conversion of FBL to myofibroblasts (Geuijen and Sonnenberg, 2002), and the interaction with CCN1/Cyr61 mediates FBL migration (Lygoe et al., 2004)
**α_v_β_6_**	FN, TGF-β1 and -β3, etc.	KCs	Regulates inflammation and KC proliferation, contributing to the basement membrane and granulation tissue remodeling (Jakhu et al., 2018)
**α_v_β_8_**	FN, and TGF-β (Lainé et al., 2021)	Dendritic cells, FBLs and ECs	Mediates TGF-β to regulate inflammation (Worthington John et al., 2015)
**α_6_β_4_**	Laminin-332, Other LM (Sehgal et al., 2006)	KC、EC	Promotes KC adhesion and migration (Geuijen and Sonnenberg, 2002); regulates angiogenesis in EC (Mercurio et al., 2001; Nikolopoulos et al., 2004)
**α_IIb_β_3_**	Fibronectin(pro), FN, CCN1/Cyr6 and CCN2/CTGF, etc (Lau, 2016; Andre et al., 2002)	Platelets	Mediates platelet aggregation in clot formation and regulates fibrin network structure and stability (antithrombotic effect) (Blue et al., 2009)
**α_4_β_7_**	VCAM, etc (Abonia et al., 2006)	Leukocytes, dendritic cells	Involved in leukocyte transport (Gubatan et al., 2021)
**α_E_β_7_**	Calcineurin	T lymphocytes, dendritic cells	Mediated leukocyte transport (Kilshaw, 1999)
**α_L_β_2_**	Lumican, etc.	Leukocytes	Mediated leukocyte extravasation through the endothelium (Tan, 2012)
**α_M_β_2_**	Fibronectin(pro), FN, CCN1/Cyr6, CCN2/CTGF, etc (Lau, 2016)	Monocytes, macrophages, NK, neutrophils, and T cells	Involved in leukocyte transport across the endothelium (Tan, 2012); complexed with uPAR and its ligand uPA to promote fibrinolysis and fibrin clot clearance by monocytes and neutrophils (Sisco et al., 2007)
**α_X_β_2_**	Fibronectin (Garnotel et al., 2000)	Monocytes, macrophages, dendritic cells, and NK	Involved in leukocyte transport (Tan, 2012)
**α_D_β_2_**	VCAM-1 and CCN1/Cyr6, etc (Lau, 2016; Grayson et al., 1998)	Macrophages, eosinophils	Involved in leukocyte transport (Tan, 2012)

FBL, fibroblasts; KC, keratin-forming cells; EC, endothelial cells; VEGF, vascular endothelial cell growth factor; FN, fibronectin; TGF-β, transforming growth factor beta; EDA, extra domain A; ADAM, a disintegrin and metalloproteinase; CCN, Cyr61-CTGF-Nov; Cyr6, cysteine-rich protein 6; VCAM, vascular cell adhesion molecule; uPAR, urokinase-type plasminogen activator receptor.

## 2 Role of integrins in bacterial infections

Prolonged inflammation may result in wounds that do not heal, such as chronic ulcers (Wang et al., 2018). The causes of chronic wounds are complex: local tissue hypoxia, wound bacterial colonization, and repetitive ischemia-reperfusion injury can all lead to chronic wounds (Mustoe et al., 2006), and inflammation due to bacterial colonization of wounds remains one of the most causes of persistent wound healing (Mustoe et al., 2006). The ECM is a non-cellular, three-dimensional macromolecular network composed of collagen, proteoglycan/glycosaminoglycan, elastin, fibronectin, laminin, and several other glycoproteins that regulate a variety of cellular functions and are essential for the maintenance of normal body homeostasis (Theocharis et al., 2016). The ECM serves as the primary microenvironment for wound healing, and integrin-mediated adhesion to the ECM may play an important role. Most chronic wounds at this stage are accompanied by bacterial infections, the most common causative agents being *S. aureus* and *P. aeruginosa* (Rhoads et al., 2012; Silva et al., 2018). Mechanisms such as the formation of bacterial biofilm, among others (Bjarnsholt, 2013; Wu et al., 2019). Such as in mouse periodontal disease (PD), bacterial biofilms inhibit β6 integrin expression and transforming growth factor-β1 signaling, leading to gingival inflammation (Uehara et al., 2022). Bacterial biofilms present in periodontal pockets inhibit αvβ6 integrin expression levels in periodontal disease and exacerbate the inflammatory response (Bi et al., 2017). Biofilm formation is tied to the regulated synthesis of extracellular matrix components (Rowan-Nash Aislinn et al., 2019), a structural group of different bacterial species that contribute to the chronicity of most wound healing, and bacteria associated with biofilms are highly resistant to antibiotics (Venkatesan et al., 2015). In addition, there are other pathogenic bacteria, such as anaerobic bacteria (Choi et al., 2019) and Streptococcus hemolytic type B (Silva et al., 2018).

### 2.1 Integrins and *S. aureus*

*S. aureus* is one of the most important human pathogens. *S. aureus* is known for its role in hospital-acquired infections and methicillin resistance and is now considered a global clinical problem (Chambers and DeLeo, 2009). This microorganism causes a variety of surface and systemic diseases and is frequently associated with oral mucositis. It is also a causative or worsening agent in various skin conditions, including atopic dermatitis, carbuncles, cellulitis, boils, hair follicles, Kawasaki syndrome, impetigo, psoriasis, and scalded skin syndrome (Morishita et al., 1999; Skov and Baadsgaard, 2000; Yarwood et al., 2000; Chiller et al., 2001; Cho et al., 2001a; Cho et al., 2001b; Breuer et al., 2002; Patel and Finlay, 2003). *S. aureus* is a major cause of wound infections and is thought to delay wound healing (Bowler et al., 2001) (**Table 2**). A prominent feature common to almost all *S. aureus* isolates is the expression of ECM-binding proteins, collectively referred to as microbial surface component recognition adhesion matrix molecules (MSCRAMMs) (Patti et al., 1994; Foster and Höök, 1998). It is possible to colonize the host by attaching to components of the ECM to initiate infection (Foster and Höök, 1998), such as cell wall-attached fibronectin-binding proteins A and B that allow bacteria to bind tightly to the ECM protein fibronectin (FN) (Flock et al., 1987; JÖNsson et al., 1991).

**Table 2:** Role of different integrins in normal wound healing (granulation tissue) and bacterial infection.

Type	Granulation tissue	Bacterial infections
α5β1	Regulates re-epithelialization and promotes migration of keratin-forming cells (Di Russo et al., 2021)	Mediating the attachment of eukaryotic cells to the extracellular matrix protein fibronectin (JÖNsson et al., 1991)
αvβ3	Regulates angiogenesis and promotes FBL proliferation (Mitchell et al., 2009)	Mediated Staphylococcus aureus bloodstream infection (Flock et al., 1987)
αvβ6	Regulates inflammation and keratin-forming cells proliferation (Jakhu et al., 2018)	Regulation of bacterial biofilms (Hynes, 1996; Mathelié-Guinlet et al., 2020)
αIIbβ3	Mediated platelet aggregation (Blue et al., 2009)	Mediated adhesion of Aureus to platelets (Miajlovic et al., 2010; Zapotoczna et al., 2013)

**Figure 4:**
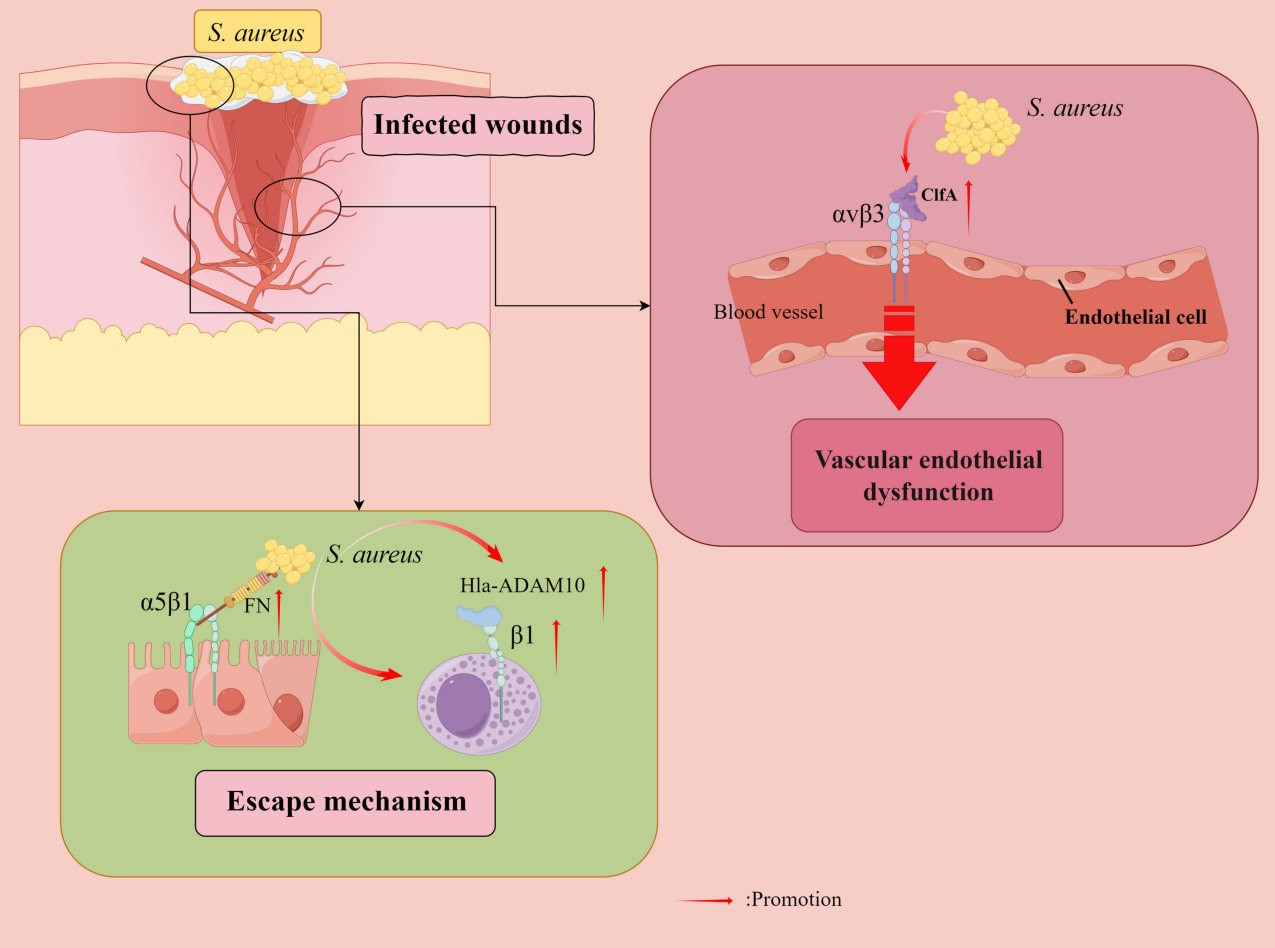
*Staphylococcus aureus (S. aureus)* evades bactericidal mechanisms. Fibronectin (FN) acts as a molecular bridge linking FnBP-expressing *S. aureus* to α5β1 integrin on the surface of human cells, tightly anchoring *S. aureus* to its eukaryotic host cells, and also facilitating microbial internalization by human epithelial and endothelial cells (ECs) and mouse fibroblasts. Furthermore, internalization of *S. aureus* by immortalized keratinocytes requires bacterial FnBPs and is mediated by the significant fibronectin-binding α5β1 integrin. *S. aureus* counteracts the extracellular bactericidal machinery of mast cells (MCs) by increasing fibronectin-binding protein expression and inducing Hla-ADAM10-mediated upregulation of β1 integrins in MCs. Vascular endothelial dysfunction is attributed to *S. aureus* aggregation factor A (ClfA) to adhere to αvβ3 integrins expressed on endothelial cells, where fibrinogen (FG) plays a key role. Direct binding of the *S. aureus* surface protein IsdB to endothelial αvβ3 integrins plays a vital role in host cell adhesion and invasion, ultimately leading to life-threatening disease. By Figdraw.

Integrin β1-containing receptors are known for their role in cell adhesion and their ability to signal the transduction of cell attachment to the ECM (Schwartz and Ginsberg, 2002). In the *in vitro* experiments, *S. aureus* can invade eukaryotic cells by indirectly engaging the β1 integrin-containing host receptor, but non-pathogenic Staphylococcus carnosus is not invasive (Agerer et al., 2003). α5β1 integrin is a vital cell surface receptor that mediates the attachment of eukaryotic cells to the ECM protein fibronectin (Hynes, 1996). FN has recently been shown to act as a molecular bridge linking FN-binding proteins (FnBP) -expressing *S. aureus* to α5β1 integrin on the surface of human cells (Joh et al., 1999). This interaction not only tightly anchors *S. aureus* to its eukaryotic host cells but also promotes the internalization of the microbe by human epithelial and endothelial cell and mouse fibroblasts (Dziewanowska et al., 1999; Sinha et al., 1999; Fowler et al., 2000; Jett Bradley and Gilmore Michael, 2002) (**Figure 4**). In addition, an *in vitro* study found that one study found that necrotizing soft tissue infections with *S. aureus* isolates showed high rates of internalization and cytotoxicity to human myocytes, and the cellular basis of the high internalization rate in myocytes was attributed to the higher expression of α5β1 integrins in myocytes (Baude et al., 2019). The ability of *S. aureus* to be internalized by and survive in host cells, such as keratinocytes, may contribute to developing persistent or chronic infections, eventually leading to deeper tissue infection or dissemination. Internalization of *S. aureus* by immortalized keratinocytes requires bacterial FnBPs and is mediated by the significant fibronectin-binding α5β1 integrin. However, unlike the internalization of immortalized keratinocytes, the internalization of *S. aureus* by native keratinocytes can occur through FnBP-dependent and non-dependent pathways (Kintarak et al., 2004). In addition, in oral infections, multi-strain oral biofilms inhibit αvβ6 integrin expression in gingival epithelial cells (Bi et al., 2017). And periodontal inflammation caused by αvβ6 integrin deficiency also resulted in significant alterations in the oral microbiome (Uehara et al., 2022). However, the second fibronectin-binding integral protein αvβ6 found on keratin-forming cells does not mediate *S. aureus* internalization (Kintarak et al., 2004).

*In vitro* infection tests have shown that *S. aureus* counteracts the extracellular bactericidal mechanism of mast cells (MCs) by increasing fibronectin-binding protein expression and inducing Hla-ADAM10 (a disintegrin and metalloproteinase 10)-mediated upregulation of β1 integrins in MCs (Goldmann et al., 2016). An experiment on mice showed that IFN-gamma intervention, partly by β1 integrins, drives enhanced antimicrobial and pro-inflammatory responses of human MCs to *S. aureus* (Swindle et al., 2015). An *in vitro* study found that a protein exported by S.aureus, α-toxin interacts with β1-integrin, a receptor for the host ECM protein, suggesting that β1-integrin may be a potential receptor for α-toxin on epithelial cells. The α-toxin inhibits *S. aureus* adhesion and internalization by interfering with integrin-mediated pathogen-host cell interactions (Liang and Ji, 2006).

In addition, an α5β1/αvβ3 integrin antagonist has been found to inhibit *S. aureus* invasion of epithelial cells (Melby et al., 2000). A study of mouse models found that vascular endothelial dysfunction was attributed to the ability of *S. aureus* aggregation factor A (ClfA) to adhere to αvβ3 integrins expressed on endothelial cell (EC), with fibrinogen (Fg) playing a pivotal role (McDonnell et al., 2016a). The direct binding of the *S. aureus* surface protein iron-regulated surface determinant B (IsdB) to EC αvβ3 integrins plays an essential role in host cell adhesion and invasion, ultimately leading to life-threatening disease (Mathelié-Guinlet et al., 2020). Therefore, αvβ3 integrin blockade represents an attractive target for treating *S. aureus* blood-borne infections. Furthermore, force-enhanced adhesion between IsdB and integrins may be one of the multiple mechanisms that have been developed by staphylococci to effectively colonize or invade their hosts while resisting the shear forces encountered in various environments after infection (Otto, 2014), and *S. aureus* can adhere to platelets through the high-affinity form of IsdB bound to the platelet integrin αIIbβ3 integrin without the need for additional ECM proteins (Miajlovic et al., 2010; Zapotoczna et al., 2013). In addition, αDβ2 integrins have been observed to have a role in Salmonella typhimurium and *S. aureus* infections (Nascimento et al., 2008).

Integrin-linked kinases and Rac1 mediate the invasion of *S. aureus* into keratinocytes, and the bacteria can invade keratinocytes via the integrin-linked kinase-Rac1 pathway. Thus, integrin-linked kinase may be a critical factor in preventing staphylococcal skin infections (Sayedyahossein et al., 2015), and therefore, this is speculated to be a biological target for the treatment of *S. aureus* infections.

### 2.2 Integrins and *P. aeruginosa*

*P. aeruginosa* is a ubiquitous gram-negative environmental bacterium that can cause serious infections in skin wounds, such as in patients with severe burns (Azzopardi et al., 2014). It can form biofilms (Mah et al., 2003) and invade and increase the host cells. *P. aeruginosa* has been shown to have the propensity to enter and colonize injured epithelial cells (Engel and Eran, 2011), and there is ample experimental evidence that loss of epithelial polarity increases the harmful effects of *P. aeruginosa* on host cells (Engel and Eran, 2011). *P. aeruginosa* has evolved ways of manipulating host epithelial cell polarity to promote infection (Engel and Eran, 2011; Tran Cindy et al., 2014). Integrins are usually restricted to the basolateral plasma membrane of epithelial cells, and when reaching the basolateral side, *P. aeruginosa* has access to integrins (Thuenauer et al., 2020). Current studies on integrin-mediated *P. aeruginosa* are mostly limited to α5β1 and αvβ5 integrins in respiratory epithelial cells (Buommino et al., 2014; Roger et al., 1999; Leroy-Dudal et al., 2004). The *P. aeruginosa* lectin the fucose-specific lectin LecB clears integrins from the surface of cells at the wound margin and blocks cell migration and wound healing dose-dependent manner (Thuenauer et al., 2020). Further studies are needed to determine the role of integrins in *P. aeruginosa* infections in infected wounds, which seems to be a clear direction for treating *P. aeruginosa* infections.

### 2.3 Integrins and other bacterial

Integrins also mediate the infectious effects of some other species of bacteria on the organism. Entry into epithelial cells and prevention of primary immune responses are prerequisites for successful colonization and subsequent infection of human hosts by *Streptococcus pyogenes* (group A streptococci, GAS). The interaction of GAS with fibrinogen promotes integrin-mediated internalization of bacteria into keratin-forming cells, and α1β1 and α5β1 integrins are the major keratin-forming cell receptors involved in this process (Siemens et al., 2011). Excessive bacterial invasion disrupts the attachment between the tooth surface and epithelium, leading to periodontitis. Integrin α5 may be involved in the invasion of aggregatibacter actinomycetemcomitans Y4 into gingival epithelial cells, and the resulting signal transduction cascade decreases cell adhesion and reduces the defensive role of gingival epithelial cells by reducing integrin expression (Kochi et al., 2017). Adhesion of *Candida albicans* germ tube human endothelial cell lines is mediated by αvβ3 and this adhesion is significantly blocked by the anti-β3 monoclonal antibody Gly-Arg-Gly-Asp-Ser-Pro (GRGDSP) peptide or heparin and completely eliminated by their combination (Santoni et al., 2001). Therefore, αvβ3 blockade may be used as one of the therapeutic options against *Candida albicans* infection. In addition, *H. pylori* induces the expression of integrin α5β1 and activates H. pylori-infected gastric epithelial cells via proteinase-activated receptor-2 (PAR2)-induced trypsin, which may play an important role in *H. pylori*-associated carcinogenesis (Seo et al., 2009).

### 2.4 Integrins and targeted therapy for bacterial infections

The integrin family, a large group of proteins in the human body, is involved in a variety of physiological processes, and for this family of proteins, we can effectively use them to regulate a number of pathophysiological processes in the organism. Based on the mechanism of integrin-mediated bacterial infection in wound healing, it appears that bacterial infection in the vast majority of cases requires the regulation of integrins. Earlier, it was found that the interaction of staphylococcal alpha toxin with α5β1 integrin and the overproduction of TNF-α may contribute to the destruction of epithelial cells during *S. aureus* infection (Liang and Ji, 2007). Recently, *S. aureus* has also been found to counteract the extracellular bactericidal mechanism of mast cell by increasing the expression of fibronectin-binding proteins and inducing Hla-ADAM10-mediated upregulation of β1 integrins in mast cell (Goldmann et al., 2016). At this point, it may be possible to effectively treat *S. aureus* infections by inhibiting targets associated with integrins. As inhibition of the major integrin αVβ3 reduces the attachment of *S. aureus* to sheared human endothelial cells (McDonnell et al., 2016b), blocking αVβ3 is an attractive target for the treatment of *S. aureus* blood-borne infections. There is evidence that alpha-melanocyte-stimulating hormone (α-MSH), a neuropeptide produced primarily by the pituitary gland but which is also produced by many extra-pituitary cells, including skin keratin-forming cells, has anti-inflammatory and antimicrobial effects and reduces the internalization of *S. aureus*. α-MSH prematurely downregulates the production of integrins such as beta1 and heat shock surface protein 70 (Donnarumma et al., 2004), to reduce infection and the inflammatory response.

In contrast, one study found that in mouse skin lacking integrin-linked kinase in the epidermis, *S. aureus* penetrated the skin 35 times more than normal skin; thus, integrin-linked kinase has potential as a targeted therapy for the prevention of *S. aureus* skin infections (Sayedyahossein et al., 2015). Fibronectin or β1 integrin-blocking antibodies completely eliminate IFN-γ-dependent *S. aureus* junctions, and IFN-γ can trigger human mast cells mediated by β1 integrins to enhance antibacterial and pro-inflammatory responses to IFN-γ-dependent *S. aureus* (Swindle et al., 2015). In these cases, increasing integrin levels requires integrin activation, and common activators such as talin, kindlin, and mechanical force (Sun et al., 2019; Lu et al., 2022). It has also been found that *P. aeruginosa* can produce the fucose-specific lectin LecB, which specifically removes integrins from the surface of cells located at the wound edge and blocks cell migration and wound healing in a dose-dependent manner (Rowan-Nash Aislinn et al., 2019; Thuenauer et al., 2020). When appropriate, integrin supplementation may antagonize this blocking effect and promote wound healing.

In clinical trials for the treatment of sepsis, cilengitide prevented ClfA from binding αVβ3 on endothelial cells, slowing infection without affecting normal endothelial cell function (McDonnell et al., 2016b). Thus, targeted inhibition of αVβ3 treatment seems to be locally applied for wound healing. The α5β1 integrin is one of the staphylococcal α-toxin receptors involved in mediating the cytotoxicity of α-toxin (Liang and Ji, 2007). α-MSH exerts a protective effect on the skin by reducing infection and inflammatory processes through the downregulation of β1 integrins (Donnarumma et al., 2004). LecB inhibitors can also be used as a treatment strategy in addition to antibiotics (Sommer et al., 2018; Thuenauer et al., 2020). In contrast, integrin receptors promoted increased binding of *S. aureus* to IFN γ-treated huMCs (Swindle et al., 2015), demonstrating the complexity of the MC response in relation to the cytokine environment. For these, there are no practical clinical studies yet, so appropriate drug development and clinical trials become a top priority for integrin-targeted therapy.

## 3 Conclusion and prospect

The integrin family is a group of functionally diverse protein families that play key roles in various physiological and pathological mechanisms by acting as a bridge between protein-cell, cell-cell, and bacterial-cell. The integrin family’s role in bacterial-cell linkage during wound healing suggests that treatment targeting integrins can effectively promote wound healing and reduce bacterial infections. However, the human body is a unified organic whole, and integrins can largely regulate the promotion of overall wound healing. Therefore, activation of integrins is preferred in most cases. At this stage, there are few studies on the activation of integrins to block bacterial infections, which is a wide research space and requires our joint efforts to fill the gap. However, in order to treat bacterial infections in pathological wound healing, the targeting of integrins needs to be context-specific and, when certain conditions allow, appropriately inhibited, and these need to be explored and evaluated more. *S. aureus* and *P. aeruginosa*, the two most common gram-positive and gram-negative bacteria in hospital-acquired infections, are reviewed in the article, which focuses on the mechanism of their invasion into the organism via integrins and provides a systematic review for the treatment of clinical bacterial infections as well as a summary of recent studies on integrins and their related derivatives as target therapeutics. In conclusion, the use of integrins as targets for blocking bacterial infections has very high potential.

## Author contributions

DY: Conceptualization, Data curation, Formal Analysis, Methodology, Writing – original draft, Writing – review & editing. ZL: Data curation, Formal Analysis, Writing – review & editing. FN: Formal Analysis, Investigation, Methodology, Writing – review & editing. YC: Conceptualization, Data curation, Formal Analysis, Funding acquisition, Investigation, Methodology, Project administration, Resources, Supervision, Validation, Visualization, Writing – review & editing.

## References

[B1] Abonia J. P. Hallgren J. Jones T. Shi T. Xu Y. Koni P.. (2006). Alpha-4 integrins and VCAM-1, but not MAdCAM-1, are essential for recruitment of mast cell progenitors to the inflamed lung. Blood 108, 1588–1594. doi: 10.1182/blood-2005-12-012781 16670268 PMC1895513

[B2] Agerer F. Michel A. Ohlsen K. Hauck C. R. (2003). Integrin-mediated Invasion of Staphylococcus aureus into Human Cells Requires Src Family Protein-tyrosine Kinases *. J. Biol. Chem. 278, 42524–42531. doi: 10.1074/jbc.M302096200 12893831

[B3] Andre P. Prasad K. S. S. Denis C. V. He M. Papalia J. M. Hynes R. O.. (2002). CD40L stabilizes arterial thrombi by a beta(3) integrin-dependent mechanism. Nat. Med. 8, 247–252. doi: 10.1038/nm0302-247 11875495

[B4] Azzopardi E. A. Azzopardi E. Camilleri L. Villapalos J. Boyce D. E. Dziewulski P.. (2014). Gram negative wound infection in hospitalised adult burn patients-systematic review and metanalysis. PloS One 9, e95042. doi: 10.1371/journal.pone.0095042 24751699 PMC3994014

[B5] Baude J. Bastien S. Gillet Y. Leblanc P. Itzek A. Tristan A.. (2019). Necrotizing Soft Tissue Infection Staphylococcus aureus but not S. pyogenes Isolates Display High Rates of Internalization and Cytotoxicity Toward Human Myoblasts. J. Infect. Dis. 220, 710–719. doi: 10.1093/infdis/jiz167 31001627

[B6] Bi J. Koivisto L. Pang A. Li M. Jiang G. Aurora S.. (2017). Suppression of αvβ6 integrin expression by polymicrobial oral biofilms in gingival epithelial cells. Sci. Rep. 7, 4411. doi: 10.1038/s41598-017-03619-7 28667248 PMC5493688

[B7] Bishop A. (2008). Role of oxygen in wound healing. J. Wound Care 17, 399–402. doi: 10.12968/jowc.2008.17.9.30937 18833899

[B8] Bjarnsholt T. (2013). The role of bacterial biofilms in chronic infections. APMIS 121, 1–58. doi: 10.1111/apm.12099 23635385

[B9] Blue R. Kowalska M. A. Hirsch J. Murcia M. Janczak C. A. Harrington A.. (2009). Structural and therapeutic insights from the species specificity and in *vivo* antithrombotic activity of a novel αIIb-specific αIIbβ3 antagonist. Blood 114, 195–201. doi: 10.1182/blood-2008-08-169243 19414864 PMC2710948

[B10] Bouzeghrane F. Mercure C. Reudelhuber T. L. Thibault G. (2004). Alpha8beta1 integrin is upregulated in myofibroblasts of fibrotic and scarring myocardium. J. Mol. Cell. Cardiol. 36, 343–353. doi: 10.1016/j.yjmcc.2003.11.007 15010273

[B11] Bowler P. G. Duerden B. I. Armstrong D. G. (2001). Wound microbiology and associated approaches to wound management. Clin. Microbiol. Rev. 14, 244–269. doi: 10.1128/cmr.14.2.244-269.2001 11292638 PMC88973

[B12] Brem H. Tomic-Canic M. (2007). Cellular and molecular basis of wound healing in diabetes. J. Clin. Invest. 117, 1219–1222. doi: 10.1172/JCI32169 17476353 PMC1857239

[B13] Breuer K. HÄussler S. Kapp A. Werfel T. (2002). Staphylococcus aureus: colonizing features and influence of an antibacterial treatment in adults with atopic dermatitis. Br. J. Dermatol. 147, 55–61. doi: 10.1046/j.1365-2133.2002.04872.x 12100185

[B14] Buommino E. Domenico M. D. Paoletti I. Fusco A. Gregorio V. D. Cozza V.. (2014). AlphaVBeta5 integrins mediates Pseudomonas fluorescens interaction with A549 cells. FBL 19, 408–415. doi: 10.2741/4215 24389192

[B15] Burkin D. J. Kaufman S. J. (1999). The α7β1 integrin in muscle development and disease. Cell Tissue Res. 296, 183–190. doi: 10.1007/s004410051279 10199978

[B16] Canchy L. Kerob D. Demessant A. L. Amici J.-M. (2023). Wound healing and microbiome, an unexpected relationship. J. Eur. Acad. Dermatol. Venereology 37, 7–15. doi: 10.1111/jdv.18854 36635613

[B17] Chambers H. F. DeLeo F. R. (2009). Waves of resistance: Staphylococcus aureus in the antibiotic era. Nat. Rev. Microbiol. 7, 629–641. doi: 10.1038/nrmicro2200 19680247 PMC2871281

[B18] Chiller K. Selkin B. A. Murakawa G. J. (2001). Skin microflora and bacterial infections of the skin. J. Invest. Dermatol. Symposium Proc. 6, 170–174. doi: 10.1046/j.0022-202x.2001.00043.x 11924823

[B19] Cho S.-H. Strickland I. Boguniewicz M. Leung D. Y. M. (2001a). Fibronectin and fibrinogen contribute to the enhanced binding of Staphylococcus aureus to atopic skin. J. Allergy Clin. Immunol. 108, 269–274. doi: 10.1067/mai.2001.117455 11496245

[B20] Cho S.-H. Strickland I. Tomkinson A. Fehringer A. P. Gelfand E. W. Leung D. Y. M. (2001b). Preferential binding of staphylococcus aureus to skin sites of th2-mediated inflammation in a murine model. J. Invest. Dermatol. 116, 658–663. doi: 10.1046/j.0022-202x.2001.01331.x 11348452

[B21] Choi Y. Banerjee A. McNish S. Couch K. S. Torralba M. G. Lucas S.. (2019). Co-occurrence of anaerobes in human chronic wounds. Microbial Ecol. 77, 808–820. doi: 10.1007/s00248-018-1231-z 30141127

[B22] da Silva R. G. Tavora B. Robinson S. D. Reynolds L. E. Szekeres C. Lamar J.. (2010). Endothelial αβ1-integrin represses pathological angiogenesis and sustains endothelial-VEGF. Am. J. Pathol. 177, 1534–1548. doi: 10.2353/ajpath.2010.100043 20639457 PMC2928983

[B23] Desgrosellier J. S. Cheresh D. A. (2010). Integrins in cancer: biological implications and therapeutic opportunities. Nat. Rev. Cancer 10, 9–22. doi: 10.1038/nrc2748 20029421 PMC4383089

[B24] Di Russo J. Young J. L. Wegner J. W. Steins T. Kessler H. Spatz J. P. (2021). Integrin α5β1 nano-presentation regulates collective keratinocyte migration independent of substrate rigidity. Elife 10, e69861. doi: 10.7554/eLife.69861 34554089 PMC8460267

[B25] Donnarumma G. Paoletti I. Buommino E. Antonietta Tufano M. Baroni A. (2004). α-MSH reduces the internalization of Staphylococcus aureus and down-regulates HSP 70, integrins and cytokine expression in human keratinocyte cell lines. Exp. Dermatol. 13, 748–754. doi: 10.1111/j.0906-6705.2004.00218.x 15560758

[B26] Dziewanowska K. Patti Joseph M. Deobald Claudia F. Bayles Kenneth W. Trumble William R. Bohach Gregory A. (1999). Fibronectin binding protein and host cell tyrosine kinase are required for internalization of staphylococcus aureus by epithelial cells. Infection Immun. 67, 4673–4678. doi: 10.1128/iai.67.9.4673-4678.1999 PMC96793 10456915

[B27] Edwards R. Harding K. G. (2004). Bacteria and wound healing. Curr. Opin. Infect. Dis. 17(2):91–6. doi: 10.1097/00001432-200404000-00004 15021046

[B28] Engel J. Eran Y. (2011). Subversion of mucosal barrier polarity by pseudomonas aeruginosa. Front. Microbiol. 2. doi: 10.3389/fmicb.2011.00114 PMC3129012 21747810

[B29] Eto K. Huet C. Tarui T. Kupriyanov S. Liu H. Z. Puzon-McLaughlin W.. (2002). Functional classification of ADAMs based on a conserved motif for binding to integrin alpha(9)beta(1) - Implications for sperm-egg binding and other cell interactions. J. OF Biol. Chem. 277, 17804–17810. doi: 10.1074/jbc.M200086200 11882657

[B30] Flock J. I. Fröman G. Jönsson K. Guss B. Signäs C. Nilsson B.. (1987). Cloning and expression of the gene for a fibronectin-binding protein from Staphylococcus aureus. EMBO J. 6, 2351–2357-2357. doi: 10.1002/j.1460-2075.1987.tb02511.x 2822388 PMC553639

[B31] Foster T. J. Höök M. (1998). Surface protein adhesins of Staphylococcus aureus. Trends Microbiol. 6, 484–488. doi: 10.1016/S0966-842X(98)01400-0 10036727

[B32] Fowler T. Wann E. R. Joh D. Johansson S. Foster T. J. Höök M. (2000). Cellular invasion by Staphylococcus aureus involves a fibronectin bridge between the bacterial fibronectin-binding MSCRAMMs and host cell β1 integrins. Eur. J. Cell Biol. 79, 672–679. doi: 10.1078/0171-9335-00104 11089915

[B33] Gál P. Varinská L. Fáber L. Novák Š Szabo P. Mitrengová P.. (2017). How signaling molecules regulate tumor microenvironment: parallels to wound repair. Molecules 22(11):1818. doi: 10.3390/molecules22111818 29072623 PMC6150347

[B34] Gardner H. Broberg A. Pozzi A. Laato M. Heino J. (1999). Absence of integrin alpha1beta1 in the mouse causes loss of feedback regulation of collagen synthesis in normal and wounded dermis. J. Cell Sci. 112, 263–272. doi: 10.1242/jcs.112.3.263 9885280

[B35] Garnotel R. Rittie L. Poitevin S. Monboisse J. C. Nguyen P. Potron G.. (2000). Human blood monocytes interact with type I collagen through alpha(x)ss(2) integrin (CD11c-CD18, gp150-95). J. OF Immunol. 164, 5928–5934. doi: 10.4049/jimmunol.164.11.5928 10820275

[B36] Geuijen C. A. W. Sonnenberg A. (2002). Dynamics of the alpha6beta4 integrin in keratinocytes. Mol. Biol. Cell 13, 3845–3858. doi: 10.1091/mbc.02-01-0601 12429829 PMC133597

[B37] Goldmann O. Tuchscherr L. Rohde M. Medina E. (2016). α-Hemolysin enhances Staphylococcus aureus internalization and survival within mast cells by modulating the expression of β1 integrin. Cell. Microbiol. 18, 807–819. doi: 10.1111/cmi.12550 26595647

[B38] Grayson M. H. van der Vieren M. Sterbinsky S. A. Michael Gallatin W. Hoffman P. A. Staunton D. E.. (1998). αdβ2 integrin is expressed on human eosinophils and functions as an alternative ligand for vascular cell adhesion molecule 1 (VCAM-1). J. Exp. Med. 188, 2187–2191. doi: 10.1084/jem.188.11.2187 9841932 PMC2212388

[B39] Grenache D. G. Zhang Z. Wells L. E. Santoro S. A. Davidson J. M. Zutter M. M. (2007). Wound healing in the α2β1 integrin-deficient mouse: altered keratinocyte biology and dysregulated matrix metalloproteinase expression. J. Invest. Dermatol. 127, 455–466. doi: 10.1038/sj.jid.5700611 17068473

[B40] Gubatan J. Keyashian K. Rubin S. J. S. Wang J. Buckman C. A. Sinha S. (2021). Anti-integrins for the treatment of inflammatory bowel disease: current evidence and perspectives. Clin. Exp. Gastroenterol. 14, 333–342. doi: 10.2147/CEG.S293272 34466013 PMC8402953

[B41] Harding K. G. Morris H. L. Patel G. K. (2002). Healing chronic wounds. BMJ 324, 160. doi: 10.1136/bmj.324.7330.160 11799036 PMC1122073

[B42] Hostetter M. K. (1996). An integrin-like protein in Candida albicans: implications for pathogenesis. Trends Microbiol. 4, 242–246. doi: 10.1016/0966-842X(96)10036-6 8795161

[B43] Høye A. M. Couchman J. R. Wewer U. M. Fukami K. Yoneda A. (2012). The newcomer in the integrin family: Integrin α9 in biology and cancer. Adv. Biol. Regul. 52, 326–339. doi: 10.1016/j.jbior.2012.03.004 22781746

[B44] Huang Z. Miao X. Patarroyo M. Nilsson G. P. Pernow J. Li N. (2016). Tetraspanin CD151 and integrin α6β1 mediate platelet-enhanced endothelial colony forming cell angiogenesis. J. Thromb. Haemostasis 14, 606–618. doi: 10.1111/jth.13248 26749288

[B45] Huhtala P. Humphries M. J. McCarthy J. B. Tremble P. M. Werb Z. Damsky C. H. (1995). Cooperative signaling by alpha 5 beta 1 and alpha 4 beta 1 integrins regulates metalloproteinase gene expression in fibroblasts adhering to fibronectin. J. Cell Biol. 129, 867–879. doi: 10.1083/jcb.129.3.867 7537277 PMC2120442

[B46] Hynes R. O. (1996). Targeted mutations in cell adhesion genes: what have we learned from them? Dev. Biol. 180, 402–412. doi: 10.1006/dbio.1996.0314 8954713

[B47] Hynes R. O. (2004). The emergence of integrins: a personal and historical perspective. Matrix Biol. 23, 333–340. doi: 10.1016/j.matbio.2004.08.001 15533754 PMC3493146

[B48] Jakhu H. Gill G. Singh A. (2018). Role of integrins in wound repair and its periodontal implications. J. Oral. Biol. Craniofacial Res. 8, 122–125. doi: 10.1016/j.jobcr.2018.01.002 PMC5993460 29892534

[B49] Jett Bradley D. Gilmore Michael S. (2002). Internalization of staphylococcus aureus by human corneal epithelial cells: role of bacterial fibronectin-binding protein and host cell factors. Infection Immun. 70, 4697–4700. doi: 10.1128/iai.70.8.4697-4700.2002 PMC128182 12117986

[B50] Joh D. Wann E. R. Kreikemeyer B. Speziale P. Höök M. (1999). Role of fibronectin-binding MSCRAMMs in bacterial adherence and entry into mammalian cells. Matrix Biol. 18, 211–223. doi: 10.1016/S0945-053X(99)00025-6 10429941

[B51] JÖNsson K. SignÄS C. MÜLler H.-P. Lindberg M. (1991). Two different genes encode fibronectin binding proteins in Staphylococcus aureus. Eur. J. Biochem. 202, 1041–1048. doi: 10.1111/j.1432-1033.1991.tb16468.x 1837266

[B52] Jun J.-I. Lau L. F. (2010). The matricellular protein CCN1 induces fibroblast senescence and restricts fibrosis in cutaneous wound healing. Nat. Cell Biol. 12, 676–685. doi: 10.1038/ncb2070 20526329 PMC2919364

[B53] Kilshaw P. J. (1999). Alpha E beta 7. Mol. Pathol. 52, 203. doi: 10.1136/mp.52.4.203 10694940 PMC395700

[B54] Kintarak S. Whawell Simon A. Speight Paul M. Packer S. Nair Sean P. (2004). Internalization of staphylococcus aureus by human keratinocytes. Infection Immun. 72, 5668–5675. doi: 10.1128/iai.72.10.5668-5675.2004 PMC517534 15385465

[B55] Kochi S. Yamashiro K. Hongo S. Yamamoto T. Ugawa Y. Shimoe M.. (2017). Aggregatibacter actinomycetemcomitans regulates the expression of integrins and reduces cell adhesion via integrin α5 in human gingival epithelial cells. Mol. Cell. Biochem. 436, 39–48. doi: 10.1007/s11010-017-3076-z 28593565

[B56] LaFlamme S. E. Auer K. L. (1996). Integrin signaling. Semin. Cancer Biol. 7, 111–118. doi: 10.1006/scbi.1996.0016 8773296

[B57] Lainé A. Labiad O. Hernandez-Vargas H. This S. Sanlaville A. Léon S.. (2021). Regulatory T cells promote cancer immune-escape through integrin αvβ8-mediated TGF-β activation. Nat. Commun. 12, 6228. doi: 10.1038/s41467-021-26352-2 34711823 PMC8553942

[B58] Lau L. F. (2016). Cell surface receptors for CCN proteins. J. Cell Communication Signaling 10, 121–127. doi: 10.1007/s12079-016-0324-z PMC4882306 27098435

[B59] Leroy-Dudal J. Gagnière H. Cossard E. Carreiras F. Di Martino P. (2004). Role of αvβ5 integrins and vitronectin in Pseudomonas aeruginosa PAK interaction with A549 respiratory cells. Microbes Infection 6, 875–881. doi: 10.1016/j.micinf.2004.05.004 15310463

[B60] Liang X. Ji Y. (2006). Alpha-toxin interferes with integrin-mediated adhesion and internalization of Staphylococcus aureus by epithelial cells. Cell. Microbiol. 8, 1656–1668. doi: 10.1111/j.1462-5822.2006.00740.x 16984420

[B61] Liang X. Ji Y. (2007). Involvement of α5β1-integrin and TNF-α in Staphylococcus aureus α-toxin-induced death of epithelial cells. Cell. Microbiol. 9, 1809–1821. doi: 10.1111/j.1462-5822.2007.00917.x 17359518

[B62] Lin C. G. Chen C.-C. Leu S.-J. Grzeszkiewicz T. M. Lau L. F. (2005). Integrin-dependent functions of the angiogenic inducer NOV (CCN3): IMPLICATION IN WOUND HEALING*. J. Biol. Chem. 280, 8229–8237. doi: 10.1074/jbc.M404903200 15611078

[B63] Lu F. Zhu L. Bromberger T. Yang J. Yang Q. Liu J.. (2022). Mechanism of integrin activation by talin and its cooperation with kindlin. Nat. Commun. 13, 2362. doi: 10.1038/s41467-022-30117-w 35488005 PMC9054839

[B64] Lygoe K. A. Norman J. T. Marshall J. F. Lewis M. P. (2004). αv integrins play an important role in myofibroblast differentiation. Wound Repair Regeneration 12, 461–470. doi: 10.1111/j.1067-1927.2004.12402.x 15260812

[B65] Mah T.-F. Pitts B. Pellock B. Walker G. C. Stewart P. S. O’Toole G. A. (2003). A genetic basis for Pseudomonas aeruginosa biofilm antibiotic resistance. Nature 426, 306–310. doi: 10.1038/nature02122 14628055

[B66] Margadant C. Raymond K. Kreft M. Sachs N. Janssen H. Sonnenberg A. (2009). Integrin α3β1 inhibits directional migration and wound re-epithelialization in the skin. J. Cell Sci. 122, 278–288. doi: 10.1242/jcs.029108 19118220

[B67] Martin P. (1997). Wound healing–aiming for perfect skin regeneration. Science 276, 75–81. doi: 10.1126/science.276.5309.75 9082989

[B68] Mathelié-Guinlet M. Viela F. Alfeo M. J. Pietrocola G. Speziale P. Dufrêne Y. F. (2020). Single-molecule analysis demonstrates stress-enhanced binding between staphylococcus aureus surface protein isdB and host cell integrins. Nano Lett. 20, 8919–8925. doi: 10.1021/acs.nanolett.0c04015 33237786

[B69] McDonnell C. J. Garciarena C. D. Watkin R. L. McHale T. M. McLoughlin A. Claes J.. (2016a). Inhibition of major integrin αVβ3 reduces Staphylococcus aureus attachment to sheared human endothelial cells. J. Thromb. Haemostasis 14, 2536–2547. doi: 10.1111/jth.13501 27606892

[B70] McDonnell C. J. Garciarena C. D. Watkin R. L. McHale T. M. McLoughlin A. Claes J.. (2016b). Inhibition of major integrin α_V_ β_3_ reduces Staphylococcus aureus attachment to sheared human endothelial cells. J. Thromb. Haemostasis 14, 2536–2547. doi: 10.1111/jth.13501 27606892

[B71] Melby A. K. Cue D. Mousa S. A. Cleary P. P. (2000). An alpha 5beta 1/alphavbeta3 integrin antagonist inhibits Staphylococcus aureus invasion of epithelial cells. Abstracts Gen. Meeting Am. Soc. Microbiol. 100, 71.

[B72] Mercurio A. M. Rabinovitz I. Shaw L. M. (2001). The α6β4 integrin and epithelial cell migration. Curr. Opin. Cell Biol. 13, 541–545. doi: 10.1016/S0955-0674(00)00249-0 11544021

[B73] Mezu-Ndubuisi O. J. Maheshwari A. (2021). The role of integrins in inflammation and angiogenesis. Pediatr. Res. 89, 1619–1626. doi: 10.1038/s41390-020-01177-9 33027803 PMC8249239

[B74] Miajlovic H. Zapotoczna M. Geoghegan J. A. Kerrigan S. W. Speziale P. Foster T. J. (2010). Direct interaction of iron-regulated surface determinant IsdB of Staphylococcus aureus with the GPIIb/IIIa receptor on platelets. Microbiology 156, 920–928. doi: 10.1099/mic.0.036673-0 20007649

[B75] Mitchell K. Szekeres C. Milano V. Svenson K. B. Nilsen-Hamilton M. Kreidberg J. A.. (2009). α3β1 integrin in epidermis promotes wound angiogenesis and keratinocyte-to-endothelial-cell crosstalk through the induction of MRP3. J. Cell Sci. 122, 1778–1787. doi: 10.1242/jcs.040956 19435806 PMC2684832

[B76] Morishita Y. Tada T. Sato A. Toi Y. Kanzaki H. Akiyama H. (1999). Possible influences of Staphylococcus aureus on atopic dermatitis — the colonizing features and the effects of staphylococcal enterotoxins. Clin. Exp. Allergy 29, 1110–1117. doi: 10.1046/j.1365-2222.1999.00593.x 10457116

[B77] Morse E. M. Brahme N. N. Calderwood D. A. (2014). Integrin cytoplasmic tail interactions. Biochemistry 53, 810–820. doi: 10.1021/bi401596q 24467163 PMC3985435

[B78] Mustoe T. A. O’Shaughnessy K. Kloeters O. (2006). Chronic wound pathogenesis and current treatment strategies: A unifying hypothesis. Plast. Reconstructive Surg. 117(7 Suppl):35S–41S. doi: 10.1097/01.prs.0000225431.63010.1b 16799373

[B79] Nakayama Y. Kon S. Kurotaki D. Morimoto J. Matsui Y. Uede T. (2010). Blockade of interaction of alpha 9 integrin with its ligands hinders the formation of granulation in cutaneous wound healing. Lab. Invest. 90, 881–894. doi: 10.1038/labinvest.2010.69 20308983

[B80] Nascimento D. O. Vieira-De-Abreu A. Pacheco P. S. Bozza P. T. Zimmerman G. Castro-Faria-Neto H. C. (2008). The role of alpha(D)beta(2) integrin in Salmonella Typhimurium and Staphylococcus aureus infection. SHOCK 29, 80–81.

[B81] Nikolopoulos S. N. Blaikie P. Yoshioka T. Guo W. Giancotti F. G. (2004). Integrin β4 signaling promotes tumor angiogenesis. Cancer Cell 6, 471–483. doi: 10.1016/j.ccr.2004.09.029 15542431

[B82] Olsson M. Järbrink K. Divakar U. Bajpai R. Upton Z. Schmidtchen A.. (2019). The humanistic and economic burden of chronic wounds: A systematic review. Wound Repair Regeneration 27, 114–125. doi: 10.1111/wrr.12683 30362646

[B83] Oommen S. Gupta S. K. Vlahakis N. E. (2011). Vascular endothelial growth factor A (VEGF-A) induces endothelial and cancer cell migration through direct binding to integrin {alpha}9{beta}1: identification of a specific {alpha}9{beta}1 binding site. J. Biol. Chem. 286, 1083–1092. doi: 10.1074/jbc.M110.175158 21071450 PMC3020715

[B84] Otto M. (2014). Physical stress and bacterial colonization. FEMS Microbiol. Rev. 38, 1250–1270. doi: 10.1111/1574-6976.12088 25212723 PMC4227950

[B85] Patel G. K. Finlay A. Y. (2003). Staphylococcal scalded skin syndrome. Am. J. Clin. Dermatol. 4, 165–175. doi: 10.2165/00128071-200304030-00003 12627992

[B86] Patti J. M. Allen B. L. McGavin M. J. Höök M. (1994). MSCRAMM-MEDIATED ADHERENCE OF MICROORGANISMS TO HOST TISSUES. Annu. Rev. Microbiol. 48, 585–617. doi: 10.1146/annurev.mi.48.100194.003101 7826020

[B87] Rhoads D. D. Cox S. B. Rees E. J. Sun Y. Wolcott R. D. (2012). Clinical identification of bacteria in human chronic wound infections: culturing vs. 16S ribosomal DNA sequencing. BMC Infect. Dis. 12, 321. doi: 10.1186/1471-2334-12-321 23176603 PMC3542000

[B88] Riederer I. Bonomo A. C. Mouly V. Savino W. (2015). Laminin therapy for the promotion of muscle regeneration. FEBS Lett. 589, 3449–3453. doi: 10.1016/j.febslet.2015.10.004 26459029

[B89] Roger P. Puchelle E. Bajolet-Laudinat O. Tournier J. M. Debordeaux C. Plotkowski M. C.. (1999). Fibronectin and alpha5beta1 integrin mediate binding of Pseudomonas aeruginosa to repairing airway epithelium. Eur. Respir. J. 13, 1301. doi: 10.1034/j.1399-3003.1999.13f14.x 10445605

[B90] Rowan-Nash Aislinn D. Korry Benjamin J. Mylonakis E. Belenky P. (2019). Cross-domain and viral interactions in the microbiome. Microbiol. Mol. Biol. Rev. 83(1):e00044-18. doi: 10.1128/mmbr.00044-00018 30626617 PMC6383444

[B91] Rusnati M. Tanghetti E. Dell’era P. Gualandris A. Presta M. (1997). alphavbeta3 integrin mediates the cell-adhesive capacity and biological activity of basic fibroblast growth factor (FGF-2) in cultured endothelial cells. Mol. Biol. Cell 8, 2449–2461. doi: 10.1091/mbc.8.12.2449 9398667 PMC25719

[B92] Santoni G. Spreghini E. Lucciarini R. Amantini C. Piccoli M. (2001). Involvement of αvβ3 integrin-like receptor and glycosaminoglycans in Candida albicans germ tube adhesion to vitronectin and to a human endothelial cell line. Microbial Pathogenesis 31, 159–172. doi: 10.1006/mpat.2001.0459 11562169

[B93] Sayedyahossein S. Xu S. X. Rudkouskaya A. McGavin M. J. McCormick J. K. Dagnino L. (2015). Staphylococcus aureus keratinocyte invasion is mediated by integrin-linked kinase and Rac1. FASEB J. 29, 711–723. doi: 10.1096/fj.14-262774 25416549

[B94] Schwartz M. A. Ginsberg M. H. (2002). Networks and crosstalk: integrin signalling spreads. Nat. Cell Biol. 4, E65–E68. doi: 10.1038/ncb0402-e65 11944032

[B95] Sehgal B. U. DeBiase P. J. Matzno S. Chew T.-L. Claiborne J. N. Hopkinson S. B.. (2006). Integrin β4 regulates migratory behavior of keratinocytes by determining laminin-332 organization*. J. Biol. Chem. 281, 35487–35498. doi: 10.1074/jbc.M606317200 16973601 PMC2820731

[B96] Senger D. R. Claffey K. P. Benes J. E. Perruzzi C. A. Sergiou A. P. Detmar M. (1997). Angiogenesis promoted by vascular endothelial growth factor: Regulation through α1β1 and α2β1 integrins. Proc. Natl. Acad. Sci. 94, 13612–13617. doi: 10.1073/pnas.94.25.13612 9391074 PMC28354

[B97] Senger D. R. Davis G. E. (2011). Angiogenesis (Cold Spring Harbor Perspectives in Biology) 3(8):a005090. doi: 10.1101/cshperspect.a005090 PMC3140681 21807843

[B98] Seo J. H. Lim J. W. Yoon J.-H. Kim H. (2009). Proteinase-activated receptor-2 mediates the expression of integrin α5 and β1 in helicobacter pylori-infected gastric epithelial AGS cells. Digestion 80, 40–49. doi: 10.1159/000216353 19478484

[B99] Shinde A. V. Kelsh R. Peters J. H. Sekiguchi K. Van De Water L. McKeown-Longo P. J. (2015). The α4β1 integrin and the EDA domain of fibronectin regulate a profibrotic phenotype in dermal fibroblasts. Matrix Biol. 41, 26–35. doi: 10.1016/j.matbio.2014.11.004 25433338 PMC4657864

[B100] Siemens N. Patenge N. Otto J. Fiedler T. Kreikemeyer B. (2011). Streptococcus pyogenes M49 Plasminogen/Plasmin Binding Facilitates Keratinocyte Invasion via Integrin-Integrin-linked Kinase (ILK) Pathways and Protects from Macrophage Killing *. J. Biol. Chem. 286, 21612–21622. doi: 10.1074/jbc.M110.202671 21521694 PMC3122219

[B101] Silva V. Marcoleta A. Silva V. Flores D. Aparicio T. Aburto I.. (2018). Prevalencia y perfil de susceptibilidad antimicrobiana en bacterias aisladas de úlceras crónicas infectadas en adultos. Rev. Chil. infectología 35, 155–162. doi: 10.4067/s0716-10182018000200155 29912253

[B102] Sinha B. François P. P. Nüße O. Foti M. Hartford O. M. Vaudaux P.. (1999). Fibronectin-binding protein acts as Staphylococcus aureus invasin via fibronectin bridging to integrin α5β1. Cell. Microbiol. 1, 101–117. doi: 10.1046/j.1462-5822.1999.00011.x 11207545

[B103] Sisco M. Chao J. D. Kim I. Mogford J. E. Mayadas T. N. Mustoe T. A. (2007). Delayed wound healing in Mac-1-deficient mice is associated with normal monocyte recruitment. Wound Repair AND REGENERATION 15, 566–571. doi: 10.1111/j.1524-475X.2007.00264.x 17650101

[B104] Skov L. Baadsgaard O. (2000). Bacterial superantigens and inflammatory skin diseases. Clin. Exp. Dermatol. 25, 57–61. doi: 10.1046/j.1365-2230.2000.00575.x 10671976

[B105] Sommer R. Wagner S. Rox K. Varrot A. Hauck D. Wamhoff E.-C.. (2018). Glycomimetic, orally bioavailable lecB inhibitors block biofilm formation of pseudomonas aeruginosa. J. Am. Chem. Soc. 140, 2537–2545. doi: 10.1021/jacs.7b11133 29272578

[B106] Sun Z. Costell M. Fässler R. (2019). Integrin activation by talin, kindlin and mechanical forces. Nat. Cell Biol. 21, 25–31. doi: 10.1038/s41556-018-0234-9 30602766

[B107] Swift M. E. Burns A. L. Gray K. L. DiPietro L. A. (2001). Age-related alterations in the inflammatory response to dermal injury. J. Invest. Dermatol. 117, 1027–1035. doi: 10.1046/j.0022-202x.2001.01539.x 11710909

[B108] Swindle E. J. Brown J. M. Rådinger M. DeLeo F. R. Metcalfe D. D. (2015). Interferon-γ enhances both the anti-bacterial and the pro-inflammatory response of human mast cells to Staphylococcus aureus. Immunology 146, 470–485. doi: 10.1111/imm.12524 26288256 PMC4610635

[B109] Takada Y. Ye X. Simon S. (2007). The integrins. Genome Biol. 8, 215. doi: 10.1186/gb-2007-8-5-215 17543136 PMC1929136

[B110] Tan S.-M. (2012). The leucocyte β2 (CD18) integrins: the structure, functional regulation and signalling properties. Bioscience Rep. 32, 241–269. doi: 10.1042/BSR20110101 22458844

[B111] Theocharis A. D. Skandalis S. S. Gialeli C. Karamanos N. K. (2016). Extracellular matrix structure. Advanced Drug Delivery Rev. 97, 4–27. doi: 10.1016/j.addr.2015.11.001 26562801

[B112] Thuenauer R. Landi A. Trefzer A. Altmann S. Wehrum S. Eierhoff T.. (2020). The pseudomonas aeruginosa lectin lecB causes integrin internalization and inhibits epithelial wound healing. mBio 11(2):e03260-19. doi: 10.1128/mbio.03260-03219 32156827 PMC7064779

[B113] Tran Cindy S. Eran Y. Ruch Travis R. Bryant David M. Datta A. Brakeman P.. (2014). Host cell polarity proteins participate in innate immunity to pseudomonas aeruginosa infection. Cell Host Microbe 15, 636–643. doi: 10.1016/j.chom.2014.04.007 24832456 PMC4062193

[B114] Uehara O. Bi J. Zhuang D. Koivisto L. Abiko Y. Häkkinen L.. (2022). Altered composition of the oral microbiome in integrin beta 6-deficient mouse. J. Oral. Microbiol. 14, 2122283. doi: 10.1080/20002297.2022.2122283 36117552 PMC9481083

[B115] Venkatesan N. Perumal G. Doble M. (2015). Bacterial resistance in biofilm-associated bacteria. Future Microbiol. 10, 1743–1750. doi: 10.2217/fmb.15.69 26517598

[B116] Vlahakis N. E. Young B. A. Atakilit A. Sheppard D. (2005). The lymphangiogenic vascular endothelial growth factors VEGF-C and -D are ligands for the integrin α9β1*. J. Biol. Chem. 280, 4544–4552. doi: 10.1074/jbc.M412816200 15590642 PMC1368959

[B117] Wang P.-H. Huang B.-S. Horng H.-C. Yeh C.-C. Chen Y.-J. (2018). Wound healing. J. Chin. Med. Assoc. 81, 94–101. doi: 10.1016/j.jcma.2017.11.002 29169897

[B118] Worthington John J. Kelly A. Smedley C. Bauché D. Campbell S. Marie Julien C.. (2015). Integrin αvβ8-mediated TGF-β Activation by effector regulatory T cells is essential for suppression of T-cell-mediated inflammation. Immunity 42, 903–915. doi: 10.1016/j.immuni.2015.04.012 25979421 PMC4448149

[B119] Wu Y.-K. Cheng N.-C. Cheng C.-M. (2019). Biofilms in chronic wounds: pathogenesis and diagnosis. Trends Biotechnol. 37, 505–517. doi: 10.1016/j.tibtech.2018.10.011 30497871

[B120] Yarwood J. M. Leung D. Y. M. Schlievert P. M. (2000). Evidence for the involvement of bacterial superantigens in psoriasis, atopic dermatitis, and Kawasaki syndrome. FEMS Microbiol. Lett. 192, 1–7. doi: 10.1111/j.1574-6968.2000.tb09350.x 11040420

[B121] Zapotoczna M. Jevnikar Z. Miajlovic H. Kos J. Foster T. J. (2013). Iron-regulated surface determinant B (IsdB) promotes Staphylococcus aureus adherence to and internalization by non-phagocytic human cells. Cell. Microbiol. 15, 1026–1041. doi: 10.1111/cmi.12097 23279065

[B122] Zeltz C. Gullberg D. (2016). The integrin–collagen connection – a glue for tissue repair? J. Cell Sci. 129, 653–664. doi: 10.1242/jcs.180992 26857815

